# Conceptual overlap of psychological constructs in low back pain

**DOI:** 10.1016/j.pain.2013.05.035

**Published:** 2013-09

**Authors:** Paul Campbell, Annette Bishop, Kate M. Dunn, Chris J. Main, Elaine Thomas, Nadine E. Foster

**Affiliations:** Arthritis Research UK Primary Care Centre, Keele University, UK

**Keywords:** Low back pain, Factor analysis, Primary care, Psychological

## Abstract

The biopsychosocial model is increasingly accepted in low back pain (LBP) research and clinical practice. In order to assess the role of psychological factors in the development and persistence of pain, a wide array of measures has been developed. Yet there is likely to be considerable conceptual overlap between such measures, and consequently, a lack of clarity about the importance of psychological factors. The aims of this study were to investigate the extent of any such overlap. An observational cohort study of 1591 LBP patients consulting in primary care completed data on a range of psychological instruments. Exploratory and confirmatory factor analyses (EFA and CFA, respectively) were carried out at the subscale level (n = 20) to investigate factor structure. The influences of the derived factors on clinical outcomes (pain intensity and self-reported disability) were then tested using linear regression. EFA yielded 4 factors, termed “Pain-related distress,” “Cognitive coping,” “Causal beliefs,” and “Perceptions of the future,” which accounted for 65.5% of the variance. CFA confirmed the validity of these factors models. The pain-related distress factor was found to have the strongest association to LBP patients’ outcomes, accounting for 34.6% of the variance in pain intensity, and 51.1% of the variance in disability. Results confirmed that considerable overlap exists in psychological measures commonly used in LBP research. Most measures tap into patients’ emotional distress. These findings help us to understand how psychological constructs relate together; implications for future research and clinical practice are discussed.

## Summary

Overlap exists between psychological measures within back pain research; the focus needs to move from single constructs to their combined influence on outcomes for patients with back pain.

## 1 Introduction

Clear evidence exists that psychological constructs such as low mood, anxiety, fear-avoidance beliefs, coping strategies, and poor self-efficacy are significant predictors of outcomes such as pain, disability, and work retention in those who have low back pain (LBP) [18], [42], [53], [54]. An extensive array of measures is currently available, specifically designed to assess these psychological constructs [18], [45]. However, there may be considerable conceptual overlap [27], and as a consequence, their distinct value as predictors of pain and associated outcomes is unclear. This standpoint is further supported in a commentary on current disputes over the relative importance of individual psychological constructs in their relation to back pain (eg, fear avoidance), where it is suggested interaction is more likely [42]. Furthermore, clinical interventions now commonly incorporate approaches that specifically attempt to elicit and address unhelpful psychological obstacles to recovery in LBP patients [24], [29], [32]. Greater clarity on information about the relatedness of psychological constructs has the potential not only to clarify the influences of psychological processes on pain perception and pain-related disability from a theoretical point of view, but also to provide a foundation for the design of more effective interventions [27], [42], [48].

One way to examine this issue is to search for an underlying common concept, or concepts, that are shared by various psychological factors. An accepted way to undertake such an examination is factor analysis. A number of previous studies have used factor analysis to investigate the relationship between psychological constructs and pain [7], [8], [35], [37], [50]. However, 3 of the previous studies included pain and disability variables within their factor analyses models [7], [8], [35], and although useful in understanding the overview of the overlap of all factors (pain, disability, and psychological), the analyses therefore did not focus solely on psychological factors. Moreover, other than the study by De Gagne et al. [8], none of the previous studies have conducted confirmatory factor analyses (CFA) to confirm the external validity of their findings [3]. Additionally, in the 2 most recent factor analyses, Mounce et al. [37] carried out a factor analysis in a nonpain population, which is not necessarily relevant to understanding of people with pain, and Rooij et al. [50] considered measures of cognitive processes related to pain (eg, fear-avoidance beliefs, coping cognitions, general self-efficacy expectations), but did not include any affective measures (eg, depression, stress, or anxiety). In addition, both Mounce et al. [37] and Rooij et al. [50] performed principal components analysis, a data reduction technique commonly used to derive the smallest number of factors, but which can produce inflated values of variance [33], and is not best suited for the exploration of factor structure [4], [6].

The aims of this study were to quantify the degree of conceptual overlap in psychological constructs related to LBP, identify the underlying factors, and investigate their clinical validity (in relationship to pain and self-reported disability).

## 2 Method

### 2.1 Design and setting

A cohort of consulters with LBP (aged between 18 and 60 years) in 8 general practices within the North Staffordshire and Cheshire area in England completed postal questionnaires about their back pain (for full details see Foster et al. [16]). Briefly, participants who consulted their doctor for LBP were identified via computerised primary care records using Read Codes (the standard method of coding and recording reasons for contact in UK general practice). Read Codes relating to LBP were used, with exclusions for “red flag” diagnoses (eg, cauda equina syndrome, significant trauma, ankylosing spondylitis, cancers). The quality and validity of the Read Code system, within these practices, is assessed annually through continual training and feedback to ensure high-level reporting of read codes during patient consultation [47]. The cohort for the present study comprised 1591 adults who had consulted for LBP and responded to the questionnaire. They included practices with a range of deprivation levels, and, given that over 96% of the UK population is registered with a primary care practice [39], they are representative of the local population.

### 2.2 Measures

Psychological measures included within this study were chosen based on previous research that has shown associations of these concepts with pain outcomes [16], [19], [27], [30], [37], [40], [42], [45], [54].

#### 2.2.1 Psychological measures

The Hospital Anxiety and Depression Scale was used to measure depressive and anxiety symptoms [57]. The measure consists of 7 questions on depressive symptoms and 7 questions on anxiety symptoms; each item is scored on a 4-point scale (0 to 3), leading to scale score ranges of 0 to 21.

Fear avoidance was measured by the Tampa Scale for Kinesiophobia, which contains 17 items about a person’s fear of movement due to pain; higher scores indicate a higher level of fear avoidance [28].

Participant coping styles were assessed using the Coping Strategies Questionnaire 24 (CSQ-24) [21]. Twenty-three items are divided into 4 scales in the questionnaire (catastrophising, diversion, re-interpretation, cognitive-coping), with higher scores indicating a higher frequency of use of the coping style.

The Pain Self Efficacy Questionnaire was used to measure the participants’ beliefs and confidence in their ability to accomplish activities and engage in activities (eg, doing household chores, being active, getting enjoyment out of things, leading a normal life) despite their level of pain [40], [41]. The measure consists of 10 items, each scored by a 6-point Likert scale, with a higher score indicating greater self-efficacy.

Illness perceptions were measured using the Illness Perception Questionnaire-Revised (IPQ-R) [36]. The IPQ-R has 12 subscales, 7 for illness perceptions (Timeline – Acute/Chronic, Consequences, Timeline – Cyclical, Emotional Representations, Illness Coherence, Personal Control, Treatment Control) and 4 on the causes of LBP (Psychological Attributions, Risk Factors, Immunity, Accident/Chance), and a final scale that accounts for the perception of the number of symptoms (IPQ-R Symptoms) that are associated with LBP. Higher scores on each subscale of the IPQ-R indicate stronger illness perceptions, with some inter-subscale items being reverse-scored.

#### 2.2.2 Pain and disability measures

Pain intensity was measured by calculating the mean of 3 numerical rating scales (0–10) for the participant’s least and usual pain intensity (in the previous 2 weeks) and current pain intensity (at the time of filling in the questionnaire). A higher score indicates a higher level of reported pain intensity [12], [56].

Disability was assessed using the 24-item Roland-Morris Disability Questionnaire [49]; it asks questions on the level of disability associated with LBP on the day of questioning and gives a score from 0 to 24 (a higher score indicates a higher level of disability).

#### 2.2.3 Additional factors

Additional factors shown to be associated with pain and disability were included [10], [13], [19]. Information was collected on age, gender, employment status (employed vs not working due to ill health or back pain, retired, unemployed, housekeeping, other), pain duration (<1 month, 1–6 months, and 7 or more months of pain duration before time of questioning), and radiating symptoms (presence of spreading pain in the legs).

### 2.3 Data analysis

To address the aims of the study, the respondents (n = 1591) were randomly allocated to 3 groups corresponding to the proposed analyses: (1) exploratory factor analysis group (n = 530); (2) confirmatory factor analysis group (n = 530); and (3) linear regression analysis group (n = 531). The random splitting of this cohort was tested for significant differences in the factors described above using analysis of variance and χ^2^ tests as appropriate. Convention related to sample sizes for factor analysis and linear regression suggests that a ratio of 5:1 to 10:1 (cases per variable or item) is acceptable [6], [26], indicating adequate sample size within these subgroups for each analysis.

#### 2.3.1 Factor analysis

It is recommended that a number of preparatory stages are completed prior to factor analysis in order to yield the best results from the data [6]. Data preparation involved missing data analysis of the scale scores, and used Missing Completely At Random (MCAR) testing (Little’s MCAR test [55]) to ascertain potential bias in data from missing responses. CFA, using AMOS version 19 (SPSS, Inc., Chicago, IL, USA), utilises “Full Information Maximum Likelihood,” and so missing data were imputed using Estimation Maximisation for the factor analysis data [3], [51], [52]. Sensitivity analysis was carried out to determine differences between nonimputed and imputed datasets. Normal data distribution was checked (Kolmogorov-Smirnov test, visual inspection of Q-Q plots, histograms), as severe nonnormally distributed data can be problematic for Maximum Likelihood factor analysis [6], though less so in large sample sizes [26]. As this study investigated conceptual overlap at a scale level, it was important to check on the reliability structures of individual items, within each scale, as imprecise results can be obtained when consideration is not given to scale structure [7]. To achieve this, Cronbach alpha values were calculated on all items, within each individual scale, to ensure internal consistency of this cohort population in comparison to the original estimates from source publications. Within factor analysis, items (in this case, scales) should correlate within the proposed factor, but not too highly, as this would suggest multicollinearity and singularity, which reduces the clarity of the unique contribution of that scale item to the factor [15]. Based on this, it is recommended that scales with very high correlations to each other should be excluded from the analysis. There are no absolute criteria in this matter, but Main [31] recommends a correlation coefficient threshold of 0.71 (corresponding to 50% of shared variance), and Field [15] recommends a threshold of 0.9. In line with these recommendations, the threshold for scale exclusion, due to between-scale correlation, was set at 0.8.

#### 2.3.2 Exploratory factor analysis (EFA)

Exploratory factor analysis (EFA) was used to investigate possible underlying factor structures within the range of psychological measures included in this study. In total, 20 scale measures were included. Principal Axis Factoring with oblique (direct oblimin) rotation was chosen based on the assumption that the factors extracted may correlate with each other. To examine whether allowing factors to correlate made a difference to the overall factor structure, sensitivity analysis was carried out using orthogonal rotation. Factor retention included both Scree plot and Guttman-Kaiser (Eigenvalues ⩾1) examination. Degree of alignment of items with factors was evaluated using a further 3 criteria; minimal factor loadings (⩾0.3), removal of scales cross-loading on multiple factors, and the requirement of at least 3 items per factor, following best practice for EFA [6]. Where Scree plot and Eigenvalues differed in factor solutions, multiple solutions were tested using fixed-factor models (ie, dependent on Scree and Eigenvalue estimations), with the best factor solution chosen from the factor structure with the highest number of loadings (⩾0.3), highest number of stable factors (ie, more than 2 items per factor, in this study scales), lowest number of cross loadings, and the lowest number of communalities (the amount of variance accounted from that item to the factor) below 0.4. Following the EFA, Cronbach alpha values were examined from the derived factors to indicate internal consistency of the scale items, with item exclusion for factors with levels below 0.7, for factors with more than 10 items, and 0.5 for factors with fewer than 10 items [43].

#### 2.3.3 Confirmatory factor analysis (CFA)

CFA was performed to determine the validity of the derived EFA model. Maximum Likelihood extraction was used to fit the data. It is recommended that factor loadings are inspected and compared to loadings within the EFA as well as inspection of a number of “fit” statistics to give indication on overall fit of the model to the data [4]. Three statistical fit indices were chosen, reflecting the consensus for CFA model fitting [2], [4]. Model checks were based on goodness-of-fit tests: Comparative Fit Index (CFI > 0.95), Goodness of Fit Index (GFI > 0.9), and Root Mean Square Error Approximation (RMSEA: <0.05, good; <0.08, acceptable; >10, poor) [2], [25]. Often, simple CFA models do not initially provide an adequate fit to the data. In such cases, inspection of the models’ modification indices can be carried out to indicate potential misspecified parameters [4]. Hence, an a priori decision was made to examine whether modifications would result in a significant improvement in model fit (significant reduction in χ^2^ value) where such modification was theoretically defensible, examples being error correlation within factors [4] or cross-loading evident from the EFA.

#### 2.3.4 Association with pain and disability

Multiple linear regression analyses were carried out to assess the cross-sectional association of the derived factors with patients’ pain and disability. Firstly, a CFA was performed on this independent group using the final factor solution (as prescribed by the initial EFA and CFA result). Model structure loadings and fit statistics were compared between this CFA and the initial CFA. Each participant was then assigned a linear score for each respective factor using the “impute factor score” command within AMOS. The factor scores were then entered as independent variables within the multiple regression models. Each factor was regressed independently on the dependent variables (pain and disability) and then with adjustment for possible confounders (age, gender, employment status, duration of pain, presence of leg pain). Each factor was then added sequentially to a regression model (one each for pain and disability). *R*^2^ change statistics were calculated to estimate the relative additional proportion of variance explained by each factor after accounting for the contribution from all confounders. To examine the influence of pain duration prior to the index consultation (<1 month of pain, 1–6 months, and 7 months or more) on the relationship of the factors to pain and disability, sensitivity analyses were carried out: with nonoverlapping 95% confidence intervals for Beta taken as support of an effect of the influence of pain duration. Analysis was carried out using SPSS version 20 and AMOS version 19 (SPSS, Inc., Chicago, IL, USA).

## 3 Results

### 3.1 Data preparation

No scale contained more than 5% of missing data, and MCAR testing showed that missing items were not dependent on each other, indicating that data were missing completely at random (χ^2^ = 36,317.85, *P* = 1.00). Given this finding, expectation maximisation imputation was performed to impute values for missing data for the variables entered into the factor analysis. No significant differences were found between the participants on any variable and so the cases could then be randomised into the 3 analysis groups (data not shown). Data distribution normality checks indicated nonnormality (significant Kolmogorov-Smirnov tests), whilst visual inspection of histograms and Q-Q plots showed this as nonsevere and the sample was large (n > 100), hence, the data are assumed to follow a normal distribution. Reliability analysis on each scale showed acceptable values for Cronbach alpha (ie, >0.7), or values comparable with the published alpha scores from the original scale measures’ publications (Table 1).

**Table 1:** Cronbach reliability values for the psychological scales.

Measure	Cronbach α (current study)	Cronbach α – comparison study (reference)	
HADS Anxiety	0.84	0.80	Mykletun et al., 2002 [38]
HADS Depression	0.85	0.76	
TSK Kinesiophobia beliefs	0.73	0.84	French et al., 2007 [17]
CSQ Catastrophising	0.86	0.85	Harland and Georgieff, 2003 [21]
CSQ Diversion	0.86	0.84	
CSQ Reinterpretation	0.83	0.77	
CSQ Cognitive coping	0.81	0.75	
Pain Self Efficacy	0.95	0.93	Nicholas et al., 2008 [41]
IPQ-R Timeline Acute/chronic	0.91	0.89	Moss-Morris et al., 2002 [36]
IPQ-R Consequences	0.87	0.84	
IPQ-R Timeline Cyclical	0.77	0.79	
IPQ-R Emotional Representation	0.88	0.88	
IPQ-R Illness coherence	0.93	0.87	
IPQ-R Personal control	0.74	0.81	
IPQ-R Treatment control	0.76	0.80	
IPQ-R Psychological Attributions	0.84	0.86	
IPQ-R Risk factors	0.70	0.77	
IPQ-R Immunity	0.76	0.67	
IPQ-R Accident/Chance	0.19	0.23	
IPQ-R Symptoms	0.95	Not tested	

HADS, Hospital Anxiety and Depression Scale; TSK, Tampa Scale for Kinesiophobia; CSQ, Coping Strategies Questionnaire; IPQ-R, Illness Perception Questionnaire Revised.

Correlations between scales were examined; no scales correlated at or above 0.8, indicating no singularity (ie, scales were not overly related to each other, signifying accepted independence for EFA).

### 3.2 Participants

The mean age of participants was 44 years, with just over 40% being male. Three-quarters of the cohort were in current employment at the time of recruitment. Fewer than 19% described a period of pain duration (persistent pain) of less than a month prior to responding, with 44% describing pain duration at 7 months or more at time of response. More than half of the cohort (59%) described pain spreading to their legs. Full characteristics of the cohort are described in Table 2.

**Table 2:** Cohort characteristics (n = 1591).

	Mean (SD)	Number (%)
Age	43.9 (10.3)	
Gender (male)		661 (41.5)
Pain duration		
Less than a month		288 (18.6)
1 to 6 months		572 (37.0)
7 or more months		685 (44.3)
Spread of pain to legs (yes)		929 (58.8)
Employed (yes)		1177 (75.1)
Pain intensity (0–10 NRS)	3.94 (2.43)	
Disability (RMDQ)	8.64 (6.04)	
Psychological scales		
HADS Anxiety	8.25 (4.55)	
HADS Depression	6.51 (4.36)	
TSK Kinesiophobia beliefs	39.72 (6.91)	
CSQ Catastrophising	9.97 (7.93)	
CSQ Diversion	15.53 (8.22)	
CSQ Reinterpretation	7.91 (6.99)	
CSQ Cognitive coping	16.27 (6.46)	
Pain Self Efficacy	37.84 (6.91)	
IPQ-R Timeline Acute/Chronic	19.66 (5.83)	
IPQ-R Consequences	17.33 (5.48)	
IPQ-R Timeline Cyclical	13.05 (3.38)	
IPQ-R Emotional Representation	16.73 (5.23)	
IPQ-R Illness coherence	13.77 (4.99)	
IPQ-R Personal control	20.49 (3.78)	
IPQ-R Treatment control	16.99 (3.33)	
IPQ-R Psychological Attributions	12.01 (4.15)	
IPQ-R Risk	15.08 (4.15)	
IPQ-R Immunity	5.36 (1.96)	
IPQ-R Accident/ Chance	5.98 (1.90)	
IPQ-R Symptoms	4.04 (2.35)	

NRS, numeric rating scale; RMDQ, Roland Morris Disability Questionnaire; HADS, Hospital Anxiety and Depression Scale; TSK, Tampa Scale for Kinesiophobia; CSQ, Coping Style Questionnaire; IPQ-R, Illness Perception Questionnaire Revised.

### 3.3 Exploratory factor analysis (EFA)

#### 3.3.1 EFA model 1

All 20 psychological measure scales were entered into a principal axis factor analysis. Bartlett’s test of sphericity (*P* < 0.001) and Kaiser-Meyer-Olkin testing (0.87) showed that factor analysis was appropriate for this dataset. Eigenvalues indicated a 6-factor model, and the Scree plot indicated a 4-factor model. The model accounted for 68.1% of the variance in the data. Oblique rotation indicated that the scales “IPQ-R Illness coherence” and “IPQ-R-Timeline cyclical” had low loadings (<0.3); 2 of the 6 factors included only 2 scales, and 5 scales cross-loaded, indicating model instability. Communality checking indicated that the scales “IPQ-R Illness coherence” and “IPQ-R Timeline cyclical” had scores below 0.4 (actually 0.14 and 0.06, respectively), and so these scales were removed from the model and the model retested.

#### 3.3.2 EFA model 2

The second model indicated a 5-factor solution using Eigenvalues and a 4-factor solution with the Scree plot, accounting for 68.3% of the variance. Within this model there were 4 scales that cross-loaded, and the scale “IPQ-R Accident/Chance” had a loading below 0.3 and a low communality (0.111). The model was retested with this item removed.

#### 3.3.3 EFA model 3

The resulting third model indicated a 4-factor solution (using both Eigenvalue and Scree plot) and accounted for 65.5% of variance in the data. This model showed the greatest stability, with only one scale (“CSQ Cognitive Coping”) cross-loading, each factor included at least 3 scales and only “IPQ-R-personal control” and “IPQ-R-symptoms” had marginal communalities (0.26 and 0.33, respectively). The cross-loading variable (“CSQ Cognitive Coping”) was retained within the model to fulfil the criteria of a stable factor (ie, more than 2 variables) following best practice guidelines [6]. A reduced-factor model was then performed (forced model of 3 factors) to see if reducing the number of factors resulted in a better-fitting model. This model showed a reduction in overall variance explained (58.1%), with an increased number of low communalities, indicating the need for further factors. Therefore, this analysis confirmed that the 4-factor model was the accepted model for this EFA (refer to Table 3). Names were then assigned to the factors: Factor 1 (Pain-related distress), Factor 2 (Causal beliefs), Factor 3 (Coping cognitions), and Factor 4 (Perceptions of the future).

**Table 3:** Final exploratory factor analysis 4-factor model.⁎

Scales	Factor 1 (Pain-related distress)	Factor 2 (Causal beliefs)	Factor 3 (Coping cognitions)	Factor 4 (Perceptions of the future)
HADS Depression	.789			
IPQ-R Emotional Representation	.707			
CSQ Catastrophising	.706			
Pain Self Efficacy	−.700			
HADS Anxiety	.650			
IPQ-R Consequences	.628			
IPQ-R Symptoms	.513			
TSK Kinesiophobia beliefs	.497			
IPQ-R Attributions		.834		
IPQ-R Immunity		.790		
IPQ-R Risk		.780		
CSQ Reinterpretation			.755	
CSQ Diversion			.716	
CSQ Cognitive Coping	−.383		.587	
IPQ-R Treatment Control				−.811
IPQ-R Timeline Acute/Chronic				.678
IPQ-R Personal Control				−.456

Explained variance (%)	33.41	13.79	10.89	7.38
Eigenvalue	5.68	2.35	1.85	1.26

HADS, Hospital Anxiety and Depression Scale; IPQ-R, Illness Perception Questionnaire Revised; CSQ, Coping Style Questionnaire; TSK, Tampa Scale for Kinesiophobia.

⁎ Factor loadings below 0.3 are not shown.

Internal consistency tests for the factors showed alpha scores as follows: Pain-related distress (0.83), Causal beliefs (0.80), Coping cognitions (0.71), and Perceptions of the future (0.68). No significant improvement in reliability resulted from the removal of any scales from the factors.

The correlation between the derived factors was then examined. “Pain related distress” correlated with “Causal beliefs” (*r* = 0.29), indicating that as distress increases, so does the patient’s belief in a cause for their back pain. “Perceptions of the future” was also correlated with “Pain related distress” (*r* = −0.59), indicating that as distress increases, the belief that a person has control over their back pain and that their back pain will improve, reduces. “Causal beliefs” also correlated with “Perceptions of the future” (*r* = −0.14), indicating persons who are less likely to attribute a cause for their back pain have an increase in a belief of a favorable outcome for their back pain. There was also correlation between “Causal beliefs” and “Coping cognitions”; the direction shows that the more a person believes in a cause for their back pain, the more they employ cognitive coping strategies (*r* = 0.12). No other factors correlated above 0.1. Both sensitivity analyses (orthogonal rotation, nonimputed dataset) indicated no marked differences in the factor model and factor structure.

### 3.4 Confirmatory factor analysis (CFA)

**Fig. 1:**
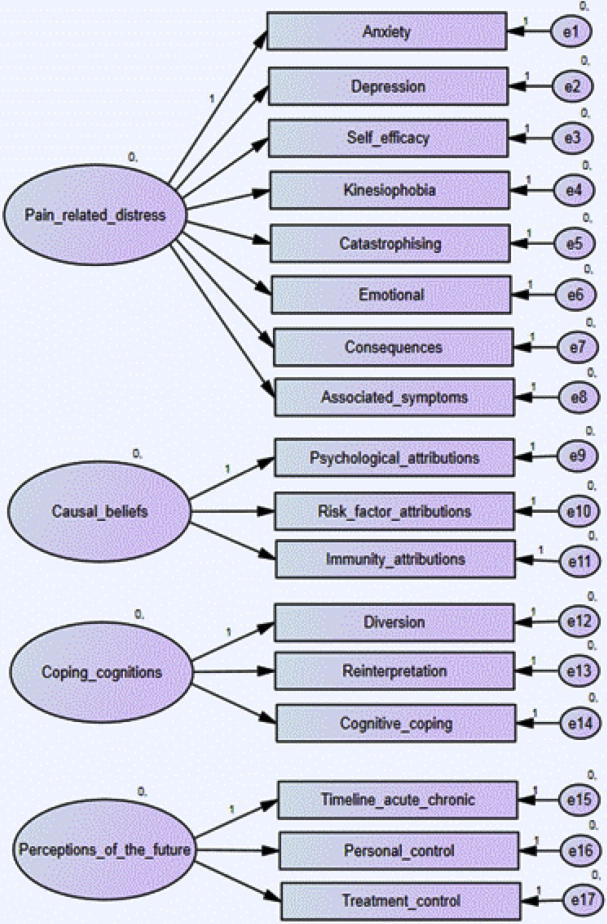
Initial confirmatory factor analysis model.

The derived EFA factor model was entered into a CFA (see Fig. 1).

Results of this initial model indicated a poor fit with the data (χ^2^ = 964.011, *df* = 119, *P* < 0.001, CFI = 0.82, GFI = 0.83, RMSEA = 0.12). As with the EFA, the second model allowed for covariance between the factors. This improved model fit from the initial CFA model, but was still judged as poor overall (χ^2^ = 754.219, *df* = 113, *P* < 0.001, CFI = 0.86, GFI = 0.86, RMSEA = 0.10).

**Fig. 2:**
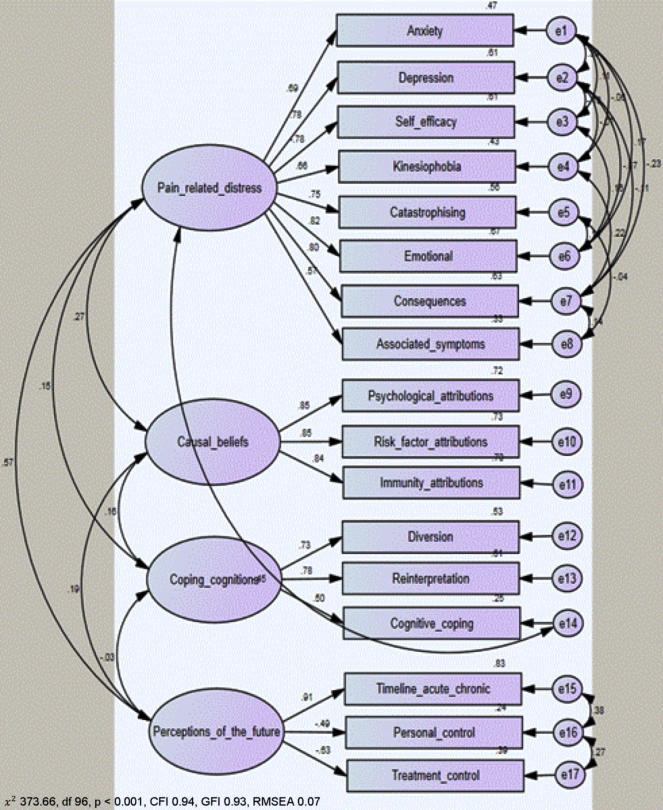
Final confirmatory factor analysis model. CFI, Comparative Fit Index; GFI, Goodness of Fit Index; RMSEA, Root Mean Square Error Approximation.

We hypothesised that there could be theoretically defensible pairwise correlations within each of the scales in each of the 4 factors. Modification indices demonstrated that a better model fit could be obtained from allowing covariance between the nonrandom errors across certain pairs of scales within the factor groups of “Pain related distress” and “Perceptions of the future.” Whilst there is shared variance for pain-related distress (ie, the factor), measures such as depression, anxiety, and emotional representation may share an affective component (not necessarily pain focused) and this may be reflected within error covariance. In the perceptions of the future factor, we predicted that respondent’s ratings of how they will be in the future (timeline acute-chronic), and the efficacy of future treatments (treatment control) would be underpinned by the person’s judgment on their level of current control and therefore, share some error variance in this regard. Results of modification showed a close to good fit (χ^2^ = 490.8, *df* = 97, *P* < 0.001, CFI = 0.92, GFI = 0.91, RMSEA = 0.09). Due to the cross-loading of the Cognitive Coping scale on the coping cognitions and pain-related distress factors in the EFA, we predicted that the addition of a correlation between the error variance of the “Coping cognitions” factor with the “Pain related distress” factor would improve overall model fit. Allowing for this additional unexplained variance showed a good fit for this model (χ^2^ = 373.66, *df* = 96, *P* < 0.001, CFI = 0.94, GFI = 0.93, RMSEA = 0.07). These results confirm the fit of the 4-factor model (Fig. 2). The standardised regression loading weights of the CFA model are shown in Table 4.

**Table 4:** Standardised regression loading weights for the confirmatory factor analysis model.

Scales	Pain-related distress	Causal beliefs	Coping cognitions	Perceptions of the future
HADS Depression	.782			
IPQ-R Emotional Representation	.816			
CSQ Catastrophising	.750			
Pain Self Efficacy	−.779			
HADS Anxiety	.686			
IPQ-R Consequences	.797			
IPQ-R Symptoms	.575			
TSK Kinesiophobia beliefs	.656			
IPQ-R Attributions		.847		
IPQ-R Immunity		.835		
IPQ-R Risk		.854		
CSQ Reinterpretation			.781	
CSQ Diversion			.731	
CSQ Cognitive Coping			.497	
IPQ-R Treatment Control				−.626
IPQ-R Timeline Acute/Chronic				.913
IPQ-R Personal Control				−.488

HADS, Hospital Anxiety and Depression Scale; IPQ-R, Illness Perception Questionnaire Revised; CSQ, Coping Style Questionnaire; TSK, Tampa Scale for Kinesiophobia.

### 3.5 Association of factors with pain and disability

The final CFA model (Fig. 2) was then applied to the linear regression subgroup. The results showed a comparable model fit to that seen in the original CFA group (χ^2^ = 325.74, *df* = 96, *P* < 0.001, CFI = 0.95, GFI = 0.94, RMSEA = 0.07) with similar standardised regression weights (no weights differed between populations by any more than 0.2). This indicated that the derived factor scores were comparable and suitable for testing in association with patients’ pain and disability.

#### 3.5.1 Pain intensity

The “Pain related distress” factor was significantly associated with pain intensity within the unadjusted, adjusted (adjustment for age, gender, employment status, duration of pain, presence of leg pain), and multivariable model (the adjusted model plus other factors). The direction of association was positive, indicating that as pain-related distress increases, so does the level of pain intensity (Table 5). The distress factor accounted for the greatest amount of variance compared to any other factor, with over 15% of the variance in pain intensity explained within the final multivariable model.

**Table 5:** Factor associations with pain intensity.

Factor	Unadjusted		Adjusted		Multivariable Model	
	β	Variance explained (%) a	β	Variance explained (%) a	β	Variance added (%) a
Pain-related distress	0.59⁎⁎	34.6%	0.45⁎⁎	15.4%	0.48⁎⁎	15.4%
Causal beliefs	0.20⁎⁎	3.9%	0.08⁎	0.6%	−0.09^∗^	0.6%
Coping cognitions	0.04	< 0.01%	0.02	< 0.01%	0.02	<0.01%
Perceptions of future	−0.37⁎⁎	13.3%	−0.22⁎⁎	3.9%	0.03	<0.01%

β, standardised beta.

⁎ *P* ⩽ 0.05.

⁎⁎ *P* ⩽ 0.001.

a *R*^2^ adjusted × 100.

The “Causal belief” factor was also statistically significant within the final multivariable model, accounting for 0.6% of the variance in pain intensity. This direction of association indicated that the more a person attributes a causal belief to their back pain, the lower pain intensity they report. Both the “Coping cognitions” and “Perceptions of the future” factors were nonsignificant within the multivariable model.

Sensitivity analysis for the relationship of factors to pain intensity based on pain duration prior to consultation shows no marked differences between the Beta coefficient and 95% confidence intervals (ie, overlap present) (data not shown). As a result, prior pain duration does not appear to markedly affect the relationship of the factors to pain intensity.

#### 3.5.2 Disability

Similar associations were found between the factors and disability, as with pain intensity (Table 6). “Pain related distress” accounted for 28% of variance in disability. The direction of association was positive, indicating increases in disability as pain-related distress increased. The “Causal belief” factor was also statistically significant, and accounted for 1.2% of the variance, indicating that patients with stronger causal attributions about their back pain report less disability. The “Perceptions of the future” factor was also statistically significant within the disability model, accounting for 0.8% of the variance. The “Coping cognition” factor was not significant at the final multivariable stage.

**Table 6:** Factor associations with disability.

Factor	Unadjusted		Adjusted		Multivariable model	
	β	Variance explained (%) a	β	Variance explained (%) a	β	Variance added (%) a
Pain-related distress	0.72⁎⁎	51.1%	0.61⁎⁎	28.0%	0.76⁎⁎	28.0%
Causal beliefs	0.26⁎⁎	6.4%	0.13⁎⁎	1.4%	−0.15⁎⁎	1.2%
Coping cognitions	0.13⁎	1.4%	0.05	0.01%	0.04	0.00%
Perceptions of future	−0.44⁎⁎	19.1%	−0.27⁎⁎	6.0%	0.13⁎	0.8%

β, standardised beta.

⁎ *P* ⩽ 0.05.

⁎⁎ *P* ⩽ 0.001.

a *R*^2^ adjusted × 100.

Again, sensitivity analyses show no marked differences by pain duration reported by the patient prior to their index consultation on the relationship of the factors to disability (data not shown).

## 4 Discussion

Our analyses show considerable overlap between regularly used psychological measures in LBP research. By far the largest overarching construct is pain-related emotional distress. This can be conceptualised as measured either directly (eg, anxiety, depression), or indirectly through the person’s current experience of pain (eg, kinesiophobia, catastrophising, emotional reaction), the person’s self-view in relation to their pain (eg, pain self-efficacy), and on the number of other symptoms, perceived to be related to the person’s back pain.

### 4.1 Comparison with previous published research

Our findings are consistent with results of previous factor analyses that have shown patients’ emotional distress as an important construct [7], [8], [35], [37], [50]. All previous factor analyses have found such a factor. Our study has extended these findings by demonstrating this within an LBP population, confirmed model fit using CFA, as well as uniquely showing the strong association of this factor with important patient-reported clinical outcomes of pain and disability.

There is also evidence of a coping cognition factor that appears distinct, with indications of similar constructs found by previous factor analysis studies [7], [35], [50]. Our results suggest that strategies that people employ to cope with back pain are distinct from the emotional experience of pain. We have shown that such a factor (termed “Coping cognitions”) has no direct relationship with pain or disability; perhaps because its content seems more to reflect cognitive processes, than emotional responses to pain. More research is needed to ascertain the relationship between such coping cognitions, pain-related emotional stress and pain outcomes, with a particular focus on the sequential processes involved. For example, using Leventhal’s Self Regulation Model [20], future research could consider what processes might lead to a perception of a health threat (physical, psychological), how this then might influence the cognitive coping processes the person adopts (adaptive, maladaptive), and what processes feedback to the individual on their sense of control and future management.

This present study, and the study by Rooij et al. [50], included the IPQ-R measure in the analyses. Both report a factor that appears to capture patients’ thoughts about the future (Rooij et al. chronic widespread pain, this study back pain). This suggests that there is a distinct construct relating to patients’ beliefs about the chronicity of their back pain (how long it will last) and the likelihood of improvement from treatments (both personal and external). However, this factor does share a strong association with “Pain related distress” (*r* = −0.59), and did not account for a credible amount of the variance (ie, <1%) in both pain and disability once adjusted. More longitudinal work is needed on the probable interaction these factors may have, such as what factors lead to the development of a belief that the patient will not improve in the future, and how then does such a belief contribute to poor outcome.

The final factor within this study’s model is “Causal beliefs.” This factor is constructed from the IPQ-R items that assess the patients’ perceptions about the likely causes of their LBP. It utilises 3 sources: psychological causes (eg, stress, worry), risk causes (eg, diet, eating habits), and immunity causes (eg, germ, virus). Interestingly, this factor had a positive relationship with pain and disability within the unadjusted and adjusted regression (ie, a higher level of attribution or belief in a specific cause was associated with higher pain and disability). However, when placed in the multivariable model, this association was reversed. This reversal may be explained by the “Causal belief” factors’ correlation (*r* = 0.22) to the “Pain related distress” factor. Further work is needed to establish how such causal beliefs are formed, particularly at the consultation stage where initial explanations might have an important impact on subsequent prognosis.

### 4.2 Implications

This study has shown that there is considerable overlap in typical measures used to assess psychological factors related to LBP. This has implications for future research and treatments that incorporate psychological factors. For example, Pincus et al. [45] found a range of studies reporting various psychological measures as potential prognostic markers in those with LBP and they suggest that most appeared to be measuring distress. Our findings support this claim. The results suggests that the emotional experience of a patient with back pain may be reflected in all of these constructs (ie, catastrophising, depression, anxiety, kinesiophobia, pain self-efficacy, and emotional reactions), as well as the emotional load of additional somatic symptoms and poorer general health associated with back pain [22]. That is not to say that these constructs are the same; our results do show variability on the loadings for the individual measures, which suggests distinctiveness in part. Given the large variability across studies on what psychological factors are significant [42], it might be more useful to combine the important psychological elements together as this may make for much stronger and potentially more clinically useful markers of those patients at higher risk of poor outcome. However, further information would be needed to combine risk identification with tailored treatment, as evidenced by Hill et al. [23], [24], who demonstrated superior clinical and economic outcomes using this approach. More research is needed to identify which psychological constructs are the most important, and for whom. Nicholas et al. (2011) suggest that relationships between psychological factors are complex, and there is a need to develop theoretical models that can incorporate likely interaction and moderation effects [42]. There is also the question of “when” psychological factors are important; 2 recent articles both suggest the fluid nature of psychological influence, whereby psychological factors may have different influences at different points in a person’s life [9], [48].

Our results are of relevance to clinical practice for those with LBP. Current psychological treatments regularly use or incorporate techniques drawn from cognitive behavioural therapy [1], [29], [30], [32]. Cognitive behavioural therapy focuses on the beliefs, feelings, and behaviours of pain patients, most often concentrated on the pain experience (eg, fear avoidance, catastrophising). Our results suggest that it may well be worthwhile giving additional attention to patients’ general emotional distress, beliefs about the cause of their illness, and perceptions on the future course of their illness.

### 4.3 Strengths and weaknesses

This is the first study to test for psychological overlap within a population of primary care patients with LBP. A major strength of this study is the large sample size, which has enabled both an EFA and CFA, allowing for confidence in the validity of our findings. Our results are based on a population with a mild to moderate level of pain intensity (mean 3.94); this is reflective of the majority of patients in other pain cohorts within primary care [14], [34] and population cohorts [5], [44], indicating generalisibility. We also included a comprehensive set of psychological measures (20 different scales) used within LBP research. Furthermore, we then had the unique opportunity of testing the association of the derived factors with clinically meaningful variables of pain and disability.

Factor analysis does, however, represent a collection of varied techniques and methods; other methods may have yielded different results. Furthermore, CFA is only a test of acceptance of the fit of a model to the data; it is plausible that other factor models fit equally or better. However, our results show similar factor structure compared to other factor studies (most often in chronic pain cohorts with variable levels of pain). Given the evidence of the amplification effects associated with pain (ie, greater pain equates with greater psychological sequelae [11]), the factor structure reported here may well be stable within different strata of pain across different pain conditions. Admittedly, even though this factor model accounted for a large proportion of variance, over 30% remained unexplained and it may be that other psychological constructs could account for this or other factors. Finally, it must be noted that all of the scales within the factors “Causal beliefs,” “Coping cognitions,” and “Perception of the future” originate from the same measure (eg, all the “Coping cognition” factor scales are from the CSQ, “Causal beliefs” and “Perception of the future” use factor scales from the IPQ-R). As a result, some of the explanation as to why these scales grouped to form factors may be a reflection of “method variance” whereby items are more likely to load together by virtue of the similarity of the measurement properties, rather than the properties of the construct itself [46], [50].

### 4.4 Conclusion

There is considerable conceptual overlap among the psychological measures regularly used in LBP research and clinical practice. The predominant factor common to these psychological measures is the patient’s overall level of pain-related emotional distress. The concept of pain-related distress is multifaceted, and further work is needed to examine what psychological concepts are key to the development and reduction of pain-related distress.

## Conflicts of interest

No author has a conflict of interest.
